# Acromicric dysplasia with stiff skin syndrome‐like severe cutaneous presentation in an 8‐year‐old boy with a missense *FBN1* mutation: Case report and literature review

**DOI:** 10.1002/mgg3.1282

**Published:** 2020-05-14

**Authors:** Tao Wang, Yuyan Yang, Qi Dong, Huijuan Zhu, Yuehua Liu

**Affiliations:** ^1^ Department of Dermatology Peking Union Medical College Hospital Chinese Academy of Medical Sciences and Peking Union Medical College Beijing China; ^2^ Peking Union Medical College and Chinese Academy of Medical Sciences Beijing China; ^3^ Department of Endocrinology Peking Union Medical College Hospital Chinese Academy of Medical Sciences and Peking Union Medical College Beijing China

**Keywords:** acromicric dysplasia, *FBN1*, stiff skin syndrome

## Abstract

**Background:**

Acromicric dysplasia is a rare heritable short‐stature syndrome with joint stiffness and varying degrees of cutaneous hardness. Stiff skin syndrome is a rare connective tissue disorder characterized by diffusely thick and hard skin from the time of birth. Heterozygous point mutations in the *FBN1* have been proposed as the predominant cause of both diseases.

**Methods:**

By performing skin biopsy, X‐ray imaging, electrocardiography, as well as whole‐genome sequencing and Sanger sequencing, we diagnosed an 8‐year‐old Chinese boy as acromicric dysplasia with severe skin stiffness caused by a heterogeneous mutation in the *FBN1*.

**Results:**

The patient presented with skin tightness, wrist and ankle stiffness, short stature and limbs, several deformed joints in the extremities, cone‐shaped epiphyses, and distinct facial features. He also had a patent foramen ovale and frequent respiratory infections. Skin biopsy showed thickened dermis and excessive collagen aggregation. Alcian blue staining indicated dermal mucopolysaccharide deposition. Mutation analysis revealed a heterozygous missense mutation, c.5243G>A (p.Cys1748Tyr), in exon 42 of the *FBN1*.

**Conclusion:**

This is a report about acromicric dysplasia with stiff skin syndrome‐like severe cutaneous presentation caused by a single hotspot mutation, further revealing the gene pleiotropy of *FBN1*.

## Graphical abstract

This study reports about the coexistence of stiff skin syndrome‐like severe cutaneous presentation and acromicric dysplasia in a single patient caused by a single hotspot mutation, further revealing the gene pleiotropy of *FBN1*.

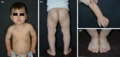

## 1 INTRODUCTION

Acromicric dysplasia (AD) (OMIM #102370) is a rare heritable severe short‐stature syndrome with the presence of joint stiffness and shortened hands and feet. Patients are born with a normal height but progressively drop down the centiles in childhood. Radiological features include delayed bone age, cone‐shaped epiphyses, and ovoid vertebral bodies. Other distinct skeletal features are internal and external notches of the femoral head and certain metacarpals. Facial abnormalities are often mild, characterized by round face, long eyelashes, bulbous nose with upturned nostrils, prominent philtrum, and thick lips with a small mouth. Frequent bronchopulmonary infections associated with tracheal problems and carpal tunnel syndromes have also been reported in some patients (Le Goff et al., 2011). Skin stiffness was seen in some of the patients, but its presentation and severity has not been well defined. Most reported patients only showed mild phenotype, but severe cases also existed. What is more, mucopolysaccharides could also accumulate in the patient's dermis, making it hard to differentiate from another genetic disease named stiff skin syndrome (Spranger, Gilbert, Tuffli, Rossiter, & Opitz, 1971). Until 2016, all heterozygous point mutations of *FBN1* resulting in AD were exclusively located in exons 41 and 42 (Sakai, Kenne, Renard, & Backer, 2016). *LTBP3* was also reported to play an etiological role in AD, but the pathomechanism behind this has remained unclear (Intarak et al., 2019).

Stiff skin syndrome (SSS) (OMIM #184900) is a rare congenital connective tissue disorder with complete penetrance. It is characterized by diffusely thickened and indurated skin from the time of birth, which limits joint mobility with flexion contractures. Some other less common features include cutaneous nodules affecting extension and flexion of the distal interphalangeal joints, and relatively short stature (Loeys et al., 2010). Histopathological assessments showed excessive aggregations of thickened collagen bundles at the dermal–epidermal junctions. Alcian blue staining was usually positive, indicating the presence of mucopolysaccharide in patients’ dermis (Geng et al., 2006; Loeys et al., 2010). Exon 37 of *FBN1* is the hotspot for heterozygous point mutations leading to SSS (Loeys et al., 2010).

Here, we report an 8‐year‐old Chinese boy diagnosed as AD related to a single heterozygous missense mutation, c.5243G>A (p.Cys1748Tyr), in exon 42 of the *FBN1*. What makes him even more special is the severe hard skin presentation. Another point mutation at the same site was reported as causing Weill–Marchesani syndrome (Cecchi et al., 2013). This article helps further illustrate the pleiotropism of *FBN1* and highlight the skin stiffness as a significant feature of AD.

### 1.1 Ethical compliance

This study was performed under the guidance of the principles of the Declaration of Helsinki. The family provided informed consent and understood that all patient samples would be used for research and genetic counseling. All regulations regarding patient enrollment, sample collection, and informed consent for the purposes of this research were followed.

## 2 CLINICAL REPORT

**Figure 1:**
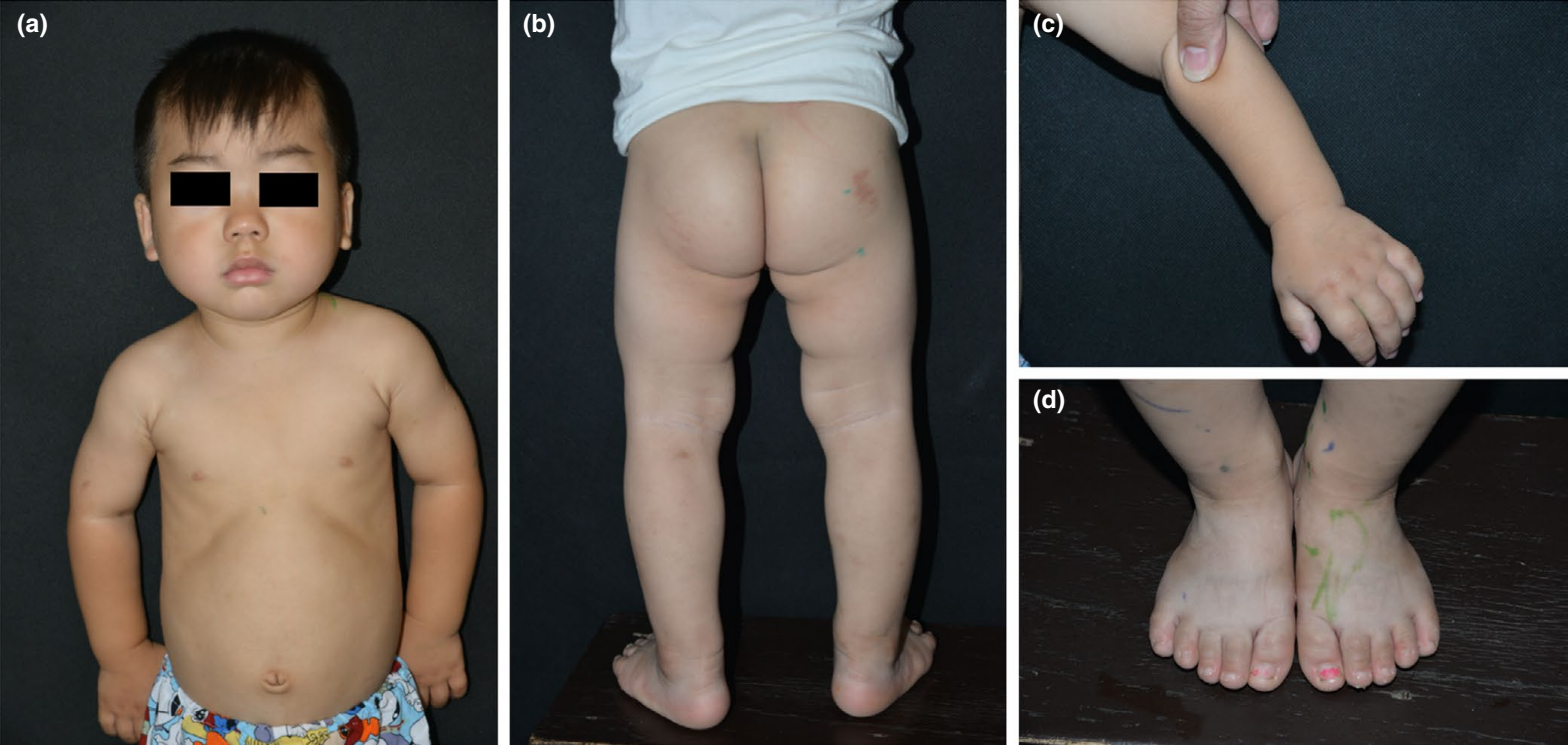
Clinical photos showing (a) and (b) skin tightness all over the body and face, facial features including epicanthus, hypotelorism and depressed nasal bridge, short limbs, as well as deformed elbows and knees; (c) and (d) wrists and ankle stiffness, joint contractures in both hands and feet and brachydactyly

**Figure 2:**
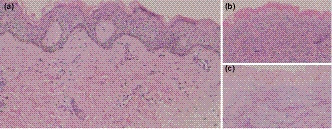
Skin biopsy showing (a) thickened dermis and excessive deposition of collagen. (b) Histopathology of the skin biopsy revealing a sparse distribution of elastin. (c) Alcian blue staining showing a mildly positive result

**Figure 3:**
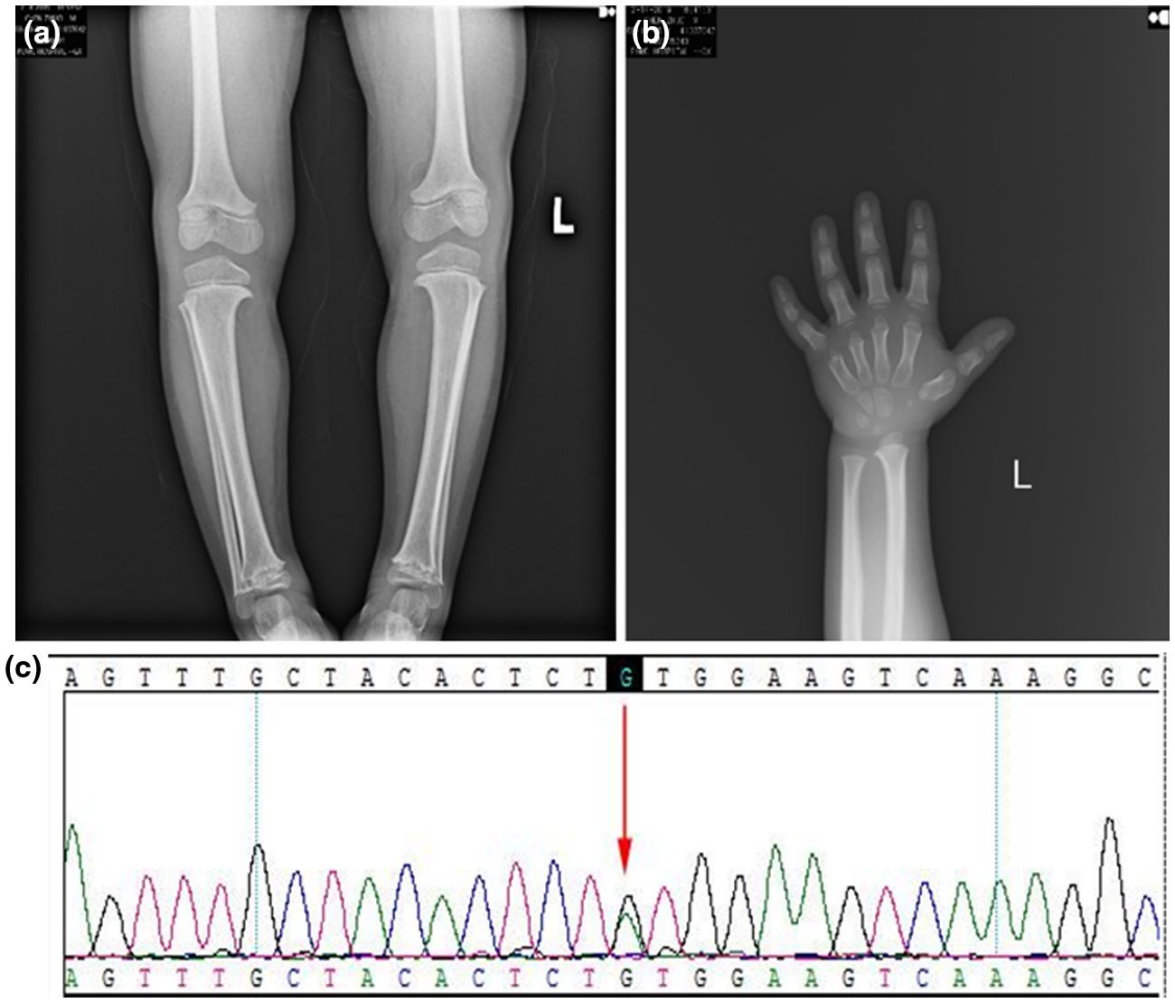
X‐ray imaging showing (a) cone‐shaped epiphysis of legs and (b) deformed interphalangeal joints. (c) Results of the mutation analysis: a heterozygous missense mutation, c.5243G>A (p.Cys1748Tyr) in exon 42 of the *FBN1*, indicated by an arrow

An 8‐year‐old boy was admitted to our clinic for hard skin all over the body and short stature, which had been present for more than 7 and 5 years, respectively (Figure 1a, b). He is the first child of a nonconsanguineous couple with no relevant family history. His mother suffered from colds four to five times during pregnancy. Apart from some Chinese herbal medicines with no teratogenic effects reported, no other medication history was mentioned. The perinatal condition was unremarkable, with birth weight of 3.5 kg (normal) and body length of 50 cm (normal). From the time of birth, skin tightness and wrist and ankle stiffness were noted (Figure 1c, d). At the age of 1 year and 3 months, short stature was noted. Skin biopsy showed mild epidermal keratosis, increased basal layer pigmentation, thickened dermis, and excessive deposition of collagen and elastin (Figure 2a, b). Alcian blue staining indicated mucopolysaccharide deposition in the dermis (Figure 2c). Since his symptoms occurred at an early age, while collagen aggregation and mucopolysaccharide deposition appeared at the same time, systemic sclerosis and scleredema could be excluded. Sclerema neonatorum was not considered because no acicular crystals were found in adipocytes. Nevertheless, only based on clinical manifestation and pathological results, SSS could not be ruled out. At the time of consultation, his skin thickening had been under little remission for 1 year without medication, although the reason for this was unknown. We inferred that environmental factors, such as food or clothes, might play a role in this transient improvement. However, the skin condition remained unchanged after then. As for the short stature, his body weight was 16.5 kg (<−3SD) and height was 99.5 cm (<−3SD). Bone age was estimated to be only 5 years old. Short limbs, cone‐shaped epiphysis of forearms and legs, significant genu varum, and deformed ankles, wrists, and interphalangeal joints, as well as brachydactyly were also noted (Figure 3a, b). There was also pronounced soft‐tissue swelling around the elbow. In addition, the patient had facial features including epicanthus, hypotelorism, and a depressed nasal bridge. Electrocardiography examination revealed patent foramen ovale. Frequent respiratory infections were also reported. Lens examination and other baseline investigations, including thyroid and function tests, appeared to be normal.

Whole‐genome sequencing and Sanger sequencing were performed on genetic DNA extracted from the peripheral blood of the patient. The results indicated a heterozygous missense mutation, c.5243G>A (p.Cys1748Tyr), in exon 42 of the *FBN1* (Figure 3c). According to this specific mutation site, we finally excluded SSS.

In conclusion, with the clinical manifestations and the substitution C1748Y, after ruling out SSS, we diagnosed this patient as having AD with extraordinarily severe skin hardness. For treatment, he was injected with recombinant human somatropin. Although no increase of IGF‐1 has been identified at the time of writing, no effect on bone growth has also yet been seen.

## 3 DISCUSSION

The *FBN1* (OMIM #184900) encodes a large cysteine‐rich modular‐secreted glycoprotein named fibrillin‐1. Its key component structures are 7 eight‐cysteinemotifs, which exhibit homology to latent TGFβ binding proteins. Principally, by recruiting ADAMTS superfamily proteins as binding proteins, fibrillin‐1 is crucial for microfibril formation and regulation (Wang, Zhang, Ye, Han, & Gu, 2014). Although the causative mechanisms of both AD and SSS remain unclear, they are believed to be related to microfibrillar malformations or dysfunctions.

AD is a rare autosomal dominant disorder, which belongs to the acromelic dysplasia group of conditions. In terms of causative mutations of AD, these were predominantly limited to mutations in exons 41 and 42 of *FBN1* (Table 1). However, *FBN1* mutations in these two exons might also cause another two short‐stature syndromes, termed geleophysic dysplasia (GD) and Weill–Marchesani syndrome (WMS) (Cecchi et al., 2013). Although these three diseases share common features, such as short stature and brachydactyly, they can still be differentiated by other features. For example, WMS patients usually present eye anomalies, including microspherophakia, ectopia lentis, glaucoma, cataracts, and myopia, which seldom appear in AD patients (Table 1) (Faivre et al., 2003). GD is more severe than AD, with additional features including tracheal stenosis and symptomatic cardiac involvement leading to early death (Jin et al., 2017). Because our patient's symptoms, especially the congenital heart disease, were milder, and he showed no eye anomalies, GD and WMS could be ruled out. Interestingly, c.5242T>C (p.Cys1748Arg) has previously been reported to cause WMS (Cecchi et al., 2013). However, the mechanism underlying this allelic condition was unclear.

**Table 1:** Genetic analysis and clinical features of *FBN1*‐mutated AD patients

Reference	Patient we reported	Moey, Flaherty, and Zankl(2019)	Jin et al.(2017)	de Bruin et al. (2016)	Wang et al.(2014)	Klein et al.(2014)	Le Goff et al. (2011)	Faivre et al.(2001)
Ethnicity	Chineses			Brazilian/African American	Chinese	French		
Age(s)	8	8	11	10/7	3.3	5.5 to 64	10 to 62	4.5 to 53
Gender	male	female	male	male/female		5 males and 4 females	12 patients	10 males and 12 females
DNA change	c.5243G>A in exon 42	c.5177G＜T in exon 41	c.5282C＜T in exon 42	c.5183C＜T in exon 41	c.5198G＜T in exon 41	heterozygous point mutations in exon41 or exon42		NA
Protein change	p.Cys1748Tyr	p.Gly1726Val	p.Thr1761Ile	p.Ala1728Val	p.Cys1733Phe			NA
Clinical Features								
Short stature	＜‐3SD	‐6.9SD	‐3.21SD	‐3.9/‐4SD	‐4.5SD	‐3 to ‐8.6SD	‐3 to ‐6SD	≤‐3SD
Joint stiffness	+		+		+	8(9)	12(12)	13(22)
Brachydactyly	+	+	+	+/+	+	9(9)	12(12)	22(22)
Delayed bone age	+	NA	+	+/+	+	8(9)	NA	22(22)
Cone‐ shaped epiphyses	+					8(9)	NA	5(22)
Notch	–					8(9)	NA	15(22)
Mild facial dysmorphism	+		+	+/+	+	9(9)	12(12)	22(22)
Skin thickness	+			−/−		3(9)	NA	NA
Cardiac abnormalities	Patent foramen ovale	Mild aortic valves stenosis, aortic incompetence, mitral valve thickening		−/−	Small atrial septal defect		none	4(22)
Respiratory complications	pulmonary infections	Upper airway resistance syndrome, airway narrowing, glottic and subglottic stenoses, severe tracheomalacia		−/−			3(12)	7(22)
Visual syndromes/eye abnormalities	−	Optic swelling, retinal nerve layer thickening, bilateral elevated discs, early morning headaches		−/−			none	8(20)
Other features	Soft tissue swelling around the elbow	Carpal tunnel syndrome	Partial growth hormone deficiency	Genu varum, hip dysplasia	Slight vertebra anomalies, hypertrophied soles and palms	1(9) genu varum, 3(9) carpal tunnel syndrome, 7(9) coxa valga	2(12) carpal tunnel syndrome, 1(12) spine stenosis	12(20) spine abnormalities, 3(22) carpal tunnel syndrome heterozygous point mutations in

Microfibrils are abundant in the extracellular matrix of the growth plate, especially in the resting and proliferative zones (Yu & Urban, 2013). TB5 domain, encoded by exons 41 and 42, has been demonstrated to interact with heparin via two binding sites to promote focal cell adhesion formation (Cain, McGovern, Baldwin, Baldock, & Kielty, 2012). Furthermore, microfibrils have been shown to regulate TGFβ/BMP bioavailability via altering their storage. Thus, fibrillin‐1 is both directly and indirectly involved in the regulation of linear bone growth (Wang et al., 2014). Interruption of the TB5 domain results in microfibril dysfunction, which might partially explain the underlying mechanism of AD. Besides, a microenvironment was predicted to be formed by domains in close proximity, including TB4 and TB5. Therefore, mutations in TB5 might perturb integrin‐binding with TB4, also leading to skin thickening. This hypothesis can partially explain the mild skin stiffness in some acromelic dysplasia patients (Sakai et al., 2016).

SSS is an autosomal dominant congenital form of scleroderma diagnosed at birth or an early age. Previously, only mutations in the sole Arg‐Gly‐Asp (RGD) motif‐encoding domain in exons 37 and 38 of FBN1 were reported to be related to SSS. Owing to disruption of this integrin‐binding TB4 domain of fibrillin‐1, excessive microfibrillar assembly and sparse elastin distribution were induced. This would impair cell matrix interactions, resulting in compensatory upregulation of αvβ3 and α5β1, leading to dermal mucopolysaccharide deposition and remarkable tissue fibrosis. TGFβ signaling was thought to play an important role in this condition, because in mouse models aggressive skin fibrosis resulting from fibrillin‐1 amino acid substitutions could be reversed by the antagonism of TGFβ (Gerber et al., 2013; Jensen, Iqbal, Bulsiewicz, & Handford, 2015; Loeys et al., 2010).

As listed in Table 1, stiff skin is not usually seen in AD patients and has no diagnostic value. We inferred that in most patients cutaneous symptoms were relatively mild compared with their bone anomalies so that they were not reported. As for the reported cases, hardly any detailed presentation or severity was mentioned. The obscure definition of skin involvement makes it hard to distinguish AD with a combination of AD and SSS when the patient born with obvious stony‐hard skin all over the body. Fortunately, no mutation hotspot overlap between these two genetic diseases has been reported yet. Whole‐genome sequencing and Sanger sequencing are strongly recommended in differentiating AD with severe hard skin and SSS.

This article reports a boy diagnosed as AD with extraordinarily severe skin stiffness due to one missense mutation, c.5242T>C (p.Cys1748Arg) in *FBN1*. This study demonstrates and expands the disease spectrum of gene polymorphism. Besides, we highlight the significance of whole‐genome sequencing and Sanger sequencing in differentiation of AD with skin symptoms and SSS.

## CONFLICTS OF INTEREST

The authors have no conflicts of interest related to the work in this manuscript.

## AUTHOR CONTRIBUTIONS

(I) Conception and design: Tao Wang, Yuehua Liu; (II) Administrative support: Huijuan Zhu, Yuehua Liu; (III) Provision of study materials or patients: Tao Wang, Qi Dong, Huijuan Zhu; (IV) Collection and assembly of data: Yuyan Yang, Tao Wang; (V) Data analysis and interpretation: Tao Wang, Yuyan Yang; (VI) Manuscript writing: Yuyan Yang, Tao Wang; (VII) Final approval of manuscript: All authors.

## Data Availability

The data used to support the findings of this study are available from the corresponding author upon request.
